# NEGATIVE AGE STEREOTYPES TEMPORARILY MAKE YOU FEEL YOUNGER BUT MAKE YOU FEEL OLDER OVER TIME

**DOI:** 10.1093/geroni/igad104.0894

**Published:** 2023-12-21

**Authors:** Anna E Kornadt, David Weiss, M Clara P de Paula Couto, Klaus Rothermund

**Affiliations:** University of Luxembourg, Esch-sur-Alzette, Luxembourg; Martin-Luther-University of Halle-Wittenberg, Halle, Sachsen-Anhalt, Germany; Friedrich Schiller University Jena, Jena, Thuringen, Germany; Friedrich Schiller University Jena, Jena, Thuringen, Germany

## Abstract

Negative age stereotypes have negative, assimilative effects on the subjective aging experience due to internalization processes, but positive contrast effects are reported as well, reflecting dissociation and downward comparisons. Our aim was thus to compare short-term and long-term consequences of age stereotypes on the subjective aging experience, to test the hypothesis that contrast effects are visible cross-sectionally, whereas internalization processes are observed when considering more long-term effects. We assessed age stereotypes and subjective age in a sample of N=459 participants (initial age range 30 – 80 years) from the aging-as-future project (Lang et al., 2022) across three consecutive measurement occasions spanning a longitudinal interval of 10 years. Short-term and long-term effects were estimated with latent change-score models by assessing effects of age stereotypes on the intercepts (cross-sectional) and on the slopes (longitudinal) of subjective age, respectively, while also taking current self-views into account. Age stereotypes had opposite effects on subjective age depending on the time frame. A cross-sectional contrast effect was found (B = -.17, SE = .05, p < .001), whereas longitudinal effects were assimilative in nature (B = .36, SE = .18, p = .04). Our findings support the time-dependent nature of effects of age stereotypes on the subjective aging experience. Negative age stereotypes can temporarily lead to a significant younger subjective age, indicating dissociation from one’s age group and downward comparison. In the long run, however, negative (positive) age stereotypes become internalized into the self-views of older people and are linked to an older (younger) subjective age.

